# A Pathology-Based Combined Model to Identify PAM50 Non-luminal Intrinsic Disease in Hormone Receptor-Positive HER2-Negative Breast Cancer

**DOI:** 10.3389/fonc.2019.00303

**Published:** 2019-04-26

**Authors:** Tomás Pascual, Miguel Martin, Aranzazu Fernández-Martínez, Laia Paré, Emilio Alba, Álvaro Rodríguez-Lescure, Giuseppe Perrone, Javier Cortés, Serafín Morales, Ana Lluch, Ander Urruticoechea, Blanca González-Farré, Patricia Galván, Pedro Jares, Adela Rodriguez, Nuria Chic, Daniela Righi, Juan Miguel Cejalvo, Giuseppe Tonini, Barbara Adamo, Maria Vidal, Patricia Villagrasa, Montserrat Muñoz, Aleix Prat

**Affiliations:** ^1^Medical Oncology Department, Hospital Clinic de Barcelona, Barcelona, Spain; ^2^SOLTI Breast Cancer Research Group, Barcelona, Spain; ^3^Medical Oncology Department, Hospital Gregorio Marañón, Universidad Complutense, Madrid, Spain; ^4^GEICAM (Spanish Breast Cancer Group), Madrid, Spain; ^5^Centro de Investigación Biomédica en Red de Oncología, CIBERONC-ISCIII, Madrid, Spain; ^6^Department of Genetics, University of North Carolina, Chapel Hill, NC, United States; ^7^Medical Oncology Department, Hospital Universitario Virgen de la Victoria, IBIMA, Málaga, Spain; ^8^Medical Oncology Department, Hospital Universitario de Elche, Elche, Spain; ^9^Department of Medicine, Università Campus Bio-Medico di Roma, Rome, Italy; ^10^IOB Institute of Oncology, Quironsalud Group, Madrid, Spain; ^11^Vall d'Hebron Institute of Oncology (VHIO), Barcelona, Spain; ^12^Medical Oncology Department, Hospital Arnau de Vilanova, Lleida, Spain; ^13^Medical Oncology Department, Hospital Clinico Universitario, Valencia, Spain; ^14^Biomedical Research Institute INCLIVA, Valencia, Spain; ^15^Department of Medicine, Universitat de València, Valencia, Spain; ^16^Department of Medical Oncology, Fundación Onkologikoa, Donostia, Spain; ^17^Department of Pathology, Hospital Clínic de Barcelona, Barcelona, Spain

**Keywords:** intrinsic subtype, non-luminal, PAM50, breast cancer, gene expression

## Abstract

**Background:** In hormone receptor-positive (HR+)/HER2-negative breast cancer, the HER2-enriched and Basal-like intrinsic subtypes are associated with poor outcome, low response to anti-estrogen therapy and high response to chemotherapy. To date, no validated biomarker exists to identify both molecular entities other than gene expression.

**Methods:** PAM50 subtyping and immunohistochemical data were obtained from 8 independent studies of 1,416 HR+/HER2-negative early breast tumors. A non-luminal disease score (NOLUS) from 0 to 100, based on percentage of estrogen receptor (ER), progesterone receptor (PR) and Ki67 tumor cells, was derived in a combined cohort of 5 studies (training dataset) and tested in a combined cohort of 3 studies. The performance of NOLUS was estimated using Area Under the ROC Curve (AUC).

**Results:** In the training dataset (*n* = 903) and compared to luminal disease, non-luminal disease had lower percentage of ER-positive cells (median 65.2 vs. 86.2%, *p* < 0.01) and PR-positive cells (33.2 vs. 56.4%, *p* < 0.01) and higher percentage of Ki67-positive cells (18.2 vs. 13.1%, *p* = 0.01). A NOLUS formula was derived: −0.45^*^ER −0.28^*^PR +0.27^*^Ki67 + 73.02. The proportion of non-luminal tumors in NOLUS-positive (≥51.38) and NOLUS-negative (<51.38) groups was 52.6 and 8.7%, respectively. In the testing dataset (*n* = 514), NOLUS was found significantly associated with non-luminal disease (*p* < 0.01) with an AUC 0.902. The proportion of non-luminal tumors in NOLUS-positive and NOLUS-negative groups was 76.9% (56.4–91.0%) and 2.6% (1.4–4.5%), respectively. The sensitivity and specificity of the pre-specified cutoff was 59.3 and 98.7%, respectively.

**Conclusions:** In the absence of gene expression data, NOLUS can help identify non-luminal disease within HR+/HER2-negative breast cancer.

## Introduction

Gene expression profiling has had a considerable impact on our understanding of hormone receptor-positive (HR+)/HER2-negative breast cancer biology (1, 2). During the last decade, two intrinsic molecular subtypes within HR+/HER2-negative disease (i.e., Luminal A and Luminal B) have been identified and intensively studied (3–5). These studies have led to well-validated prognostic gene expression-based tests such as Prosigna (6), OncotypeDX (7), MammaPrint (8), Breast Cancer Index (9),and EndoPredict (10). The implementation of these 4 platforms in the clinical practice has been essential in order to identify a subset of Luminal A tumors that can safely spare (neo)adjuvant chemotherapy treatments because of their good prognostic (11–13).

At the same time, cumulative evidence from recent studies suggests that 5–30% of HR+/HER2-negative tumors are not Luminal A or B by gene expression and fall into the HER2-enriched (HER2-E) and Basal-like categories (14). From a clinical perspective, these non-luminal tumors have been associated with low estrogen dependency (15–17), high chemo-sensitivity (18–20), potential lower activity of CDK4/6 inhibitors (21, 22) and poor outcome in both early and the advanced/metastatic breast cancer (22–24). Thus, clinical utility of the identification of the two non-luminal subtypes within HR+/HER2-negative disease is now being pursued.

In this study, we sought to validate a simple pathology-based model to help clinicians and researchers identify non-luminal disease within HR+/HER2-negative breast cancer in the absence of gene expression data.

## Materials and Methods

### Study Design

PAM50 gene expression and pathology-based data from 1,416 HR+/HER2-negative early breast tumors were obtained from 8 independent studies that are summarized in Table 1 (20, 25–30). The GEICAM/9906 is a phase III adjuvant trial in women with lymph node-positive disease that compared treatment with fluorouracil, epirubicin, and cyclophosphamide (FEC) or with FEC followed by weekly paclitaxel (FEC-P) (25). A total of 531 HR+/HER2-negative tumor samples were analyzed (26). SOLTI-1007 NeoEribulin trial is a neoadjuvant trial within HER2-negative breast cancer, where patients were treated with eribulin monotherapy for 4 cycles (20). A total of 93 HR+/HER2-negative baseline tumor samples were analyzed. Pre-operative endocrine treatment (PETx) cohort is a retrospective Spanish registry of 56 patients with HR+/HER2-negative disease treated with neoadjuvant endocrine therapy. From this study, baseline samples were analyzed (30). From GEICAM/2009-03_CONVERTHER, a study that aimed to compared pathology and gene expression data between primary and metastatic tumor samples, we obtained 50 HR+/HER2-negative primary tumor samples (28, 31). GEICAM/2012-09 is a prospective study of the Spanish Breast Cancer Research Group to characterize the impact of Prosigna assay in adjuvant treatment decision of postmenopausal patients with HR+/HER2-negative breast cancer without nodal involvement (27). A total of 174 primary tumor samples were included. Hospital Clinic of Barcelona (HCB) cohort is a consecutive series of 194 tumor samples where Prosigna has been performed as routine clinical care (29). Università Campus Bio-Medico di Roma (CBM) cohort is a consecutive series of 145 tumor samples where Prosigna has been performed as routine clinical care (29). Instituto de Investigación Biomédica de Málaga (IBIMA) cohort includes 180 HR+/HER2-negative baseline tumors treated with neoadjuvant chemotherapy as routine clinical practice (18).

**Table 1:** Main features of the cohorts analyzed in this study.

	**GEICAM/9906**	**SOLTI-Neoeribulin**	**PETx**	**GEICAM/2009-03**	**GEICAM/2012-09**	**HCB**	**IBIMA**	**CBM**
Dataset	Training	Training	Training	Training	Training	Testing	Testing	Testing
N	531	93	56	50	173	194	176	144
IHC	Centralized	Local	Local	Centralized	Centralized	Centralized	Centralized	Centralized
Platform	qRT-PCR	nCounter	nCounter	nCounter	nCounter	nCounter	nCounter	nCounter
PAM50 non-luminal disease (%)	77 (14.5)	12 (12.9)	3 (5.3)	7(14)	5 (2.9)	7 (3.6)	21 (11.9)	5 (3.5)
HER2-E (%)	71 (13.4)	1 (1.1)	3 (5.3)	6 (12)	4 (2.3)	4 (2.1)	7 (4.0)	3 (2.1)
Basal-like (%)	6 (1.3)	11 (11.8)	0	1 (2)	1 (0.6)	3 (1.5)	14 (7.9	2 (1.4)

### Pathology-Based Data

The formalin-fixed paraffin-embedded tumor samples analyzed met the following criteria: (1) they were obtained from untreated primary tumors, (2) estrogen receptor (ER) and progesterone receptor (PR) positivity was defined as >1% positive tumor cells according to the ASCO/CAP guidelines (32), (3) HER2-negativity was defined according to the 2013 ASCO/CAP guidelines (33). Ki67 IHC was quantified according to the 2011 Guidelines developed by the International Ki67 in Breast Cancer working group (34).

### PAM50 Intrinsic Subtyping

A research-based PAM50 subtyping assay was performed using the nCounter as previously described (24, 35, 36), except in GEICAM/9906, where a research-based PAM50 qRT-PCR-based assay was used, and GEICAM/2012-09, HCB, IBIMA, and CBM datasets, which used the standardized and commercial version of the PAM50 assay (i.e., Prosigna®). Original subtype calls obtained from each study were used. From the research-based PAM50 version, we eliminated any tumor samples identified as normal-like.

### Non-luminal Disease Score (NOLUS)

A combined score to identify non-luminal disease by PAM50 was derived from a combined dataset of 5 studies (i.e., training dataset) using ER, PR, and Ki67 levels (i.e., % of positive tumor cells). The optimal cutoff was defined as the point with the most significant (Fisher's exact test) split between Luminal and non-Luminal disease. Once NOLUS was developed, the final model and cutoff were tested in 513 HR+/HER2-negative tumors (i.e., testing set) from 3 independent databases: HCB, IBIMA, and CBM studies.

### Statistical Analysis

Univariate and multivariable logistic regression analyses were done to investigate the association of each IHC biomarkers with non-luminal disease. Odds ratios (ORs) and 95% confidence intervals (CI) were calculated for each variable. The performance of NOLUS was estimated using Area Under the ROC Curve (AUC). 10-fold cross-validation was conducted (37). The significance level was set to a two-sided α of 0.05. We used R version 3.3.1 for all the statistical analyses.

## Results

### Proportion of Non-luminal Disease Within HR+/HER2-Negative Breast Cancer

A total of 903 HR+/HER2-negative tumor samples from 5 studies were used as the training dataset (Table 1). In this cohort, non-luminal subtypes represented 11.6% (105/903) of the cases, ranging from 2.9% in GEICAM/2012-09 to 14.5% in GEICAM/9906. As expected, a relationship between chemotherapy cohorts and higher proportion of non-luminal disease was found. The 3 chemotherapy cohorts had proportions of non-luminal disease >10%, whereas the 2 hormonotherapy cohorts, the Spanish neoadjuvant endocrine therapy registry (PETx) and the GEICAM/2012-09 prospective study, had 2.9 and 5.4% of non-luminal tumors, respectively.

### Expression of ER, PR, and Ki67 in Non-luminal Disease in the Training Dataset

**Figure 1:**
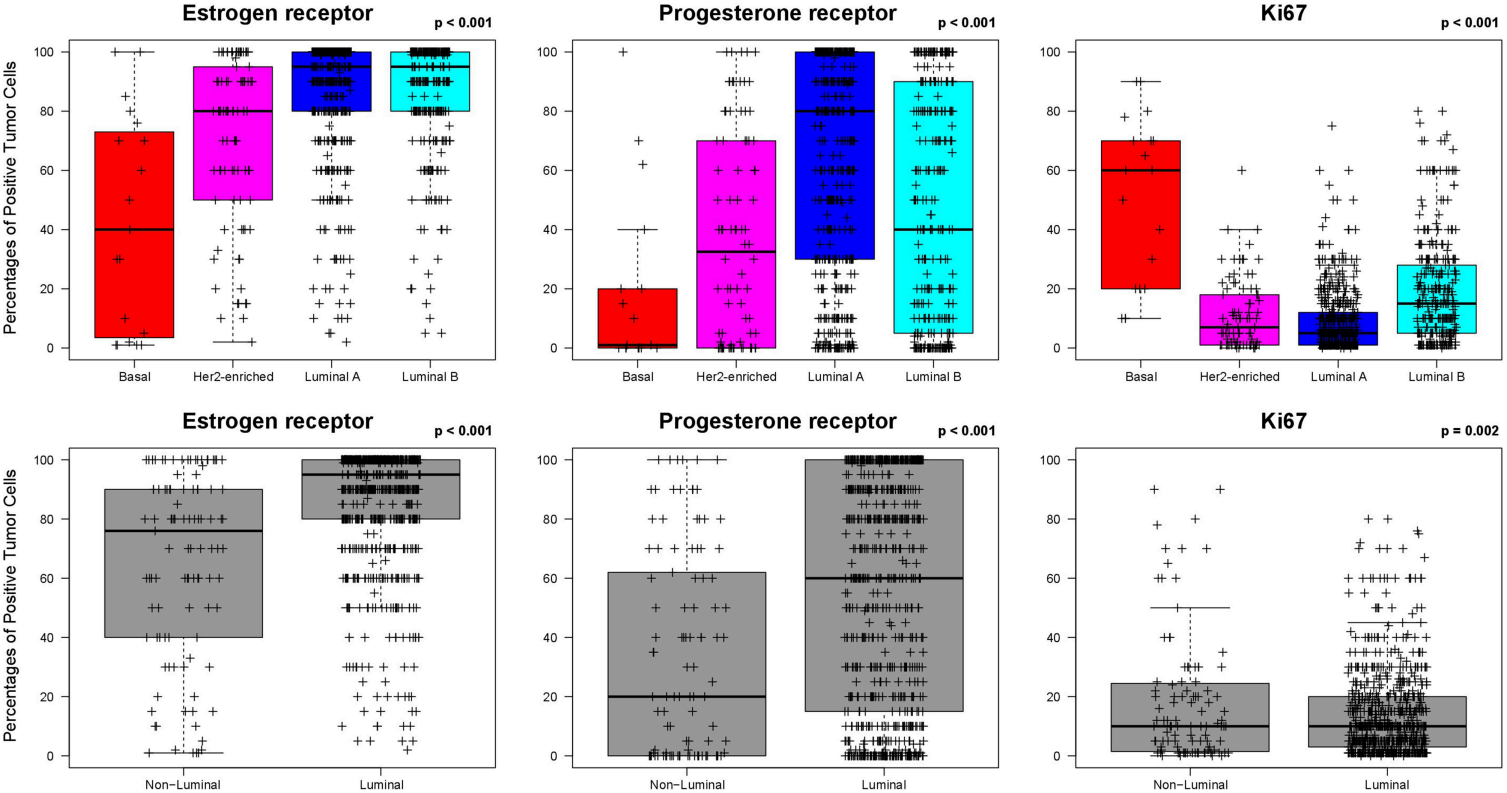
Levels of estrogen receptor (ER), progesterone receptor (PR) and Ki67-positive cells across the PAM50 intrinsic subtypes in HR+/HER2-negative breast cancer. Data was obtained from the training dataset.

ER, PR, and Ki67 were found differentially expressed (*p* < 0.001) between PAM50 luminal (*n* = 798) and non-luminal (*n* = 105) disease. Non-luminal disease had lower percentage of ER-positive cells (median 65.2 vs. 86.2%, *p* < 0.01) and PR-positive cells (33.2 vs. 56.4%, *p* < 0.01) and higher percentage of Ki67-positive cells (18.2 vs. 13.1%, *p* = 0.01) compared to luminal disease (Figure 1).

### Predicting Non-luminal Disease Using ER, PR, and Ki67

To evaluate if ER, PR, and Ki67 (measured as continuous variables) provide independent information from each other regarding the identification of non-luminal disease, a multivariable logistic regression model was applied (Table S1). Interestingly, the expression of the 3 biomarkers was found independently associated with non-luminal disease. Using this multivariable result, we developed a combined score, called non-luminal disease score (NOLUS), that weights the value of each biomarker to identify non-luminal disease. The estimated coefficient of each variable in the logistic model was used to derive NOLUS (0–100) = −0.45^*^ER% −0.28^*^PR% + 0.27^*^Ki67% + 73, where ER, PR, and Ki67 are measured as continuous variables based on the percentage of positive tumor cells by immunohistochemistry.

**Figure 2:**
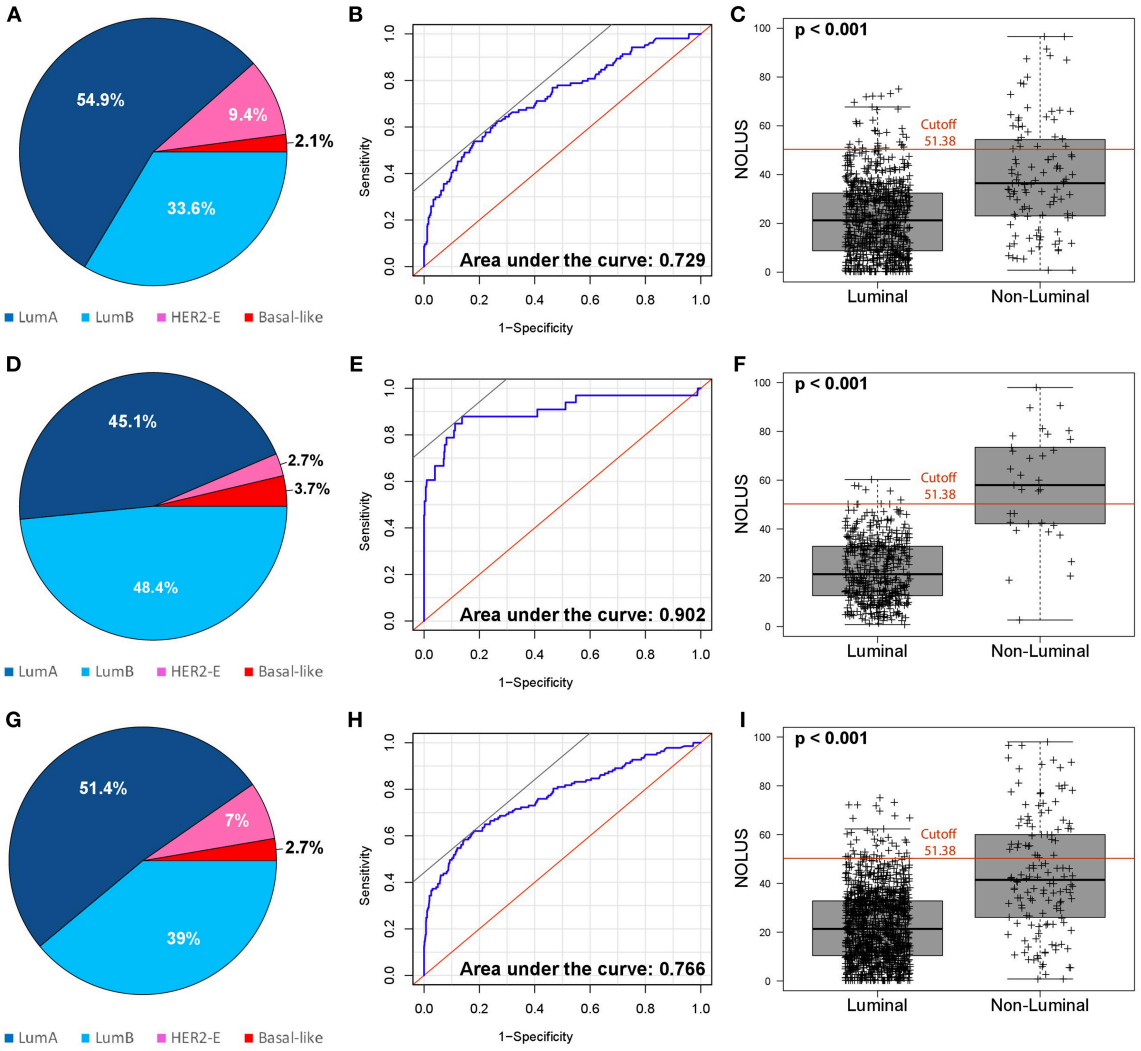
Performance of NOLUS score to predict non-luminal subtype. **(A)** Distribution of the intrinsic subtypes in the training dataset; **(B)** NOLUS score to predict non-luminal disease in the training dataset; **(C)** Expression of NOLUS in luminal vs. non-luminal tumors with the pre-specified cutoff in the training dataset; **(D)** Distribution of the intrinsic subtypes in testing dataset; **(E)** NOLUS score to predict non-luminal disease in the testing dataset; **(F)** Expression of NOLUS in luminal vs. non-luminal tumors with the pre-specified cutoff in the testing dataset; **(G)** Distribution of the intrinsic subtypes in all patients; **(H)** NOLUS score to predict non-luminal subtype in all patients; **(I)** Expression of NOLUS in luminal vs. non-luminal tumors with the pre-specified cutoff in all patients.

Next, we identified a NOLUS cutoff to identify non-luminal disease based on the most significant split using a Fisher's exact test. Using this cutoff of 51.38, the proportion of NOLUS-positive (≥51.38) tumors and NOLUS-negative (<51.38) tumors was 6.3 and 93.7%, respectively. In addition, the proportion of non-luminal tumors in NOLUS-positive and NOLUS-negative groups was 52.6% (95% CI 38.9–66.0) and 8.7% (95 CI 6.97–10.77), respectively (*p* < 0.001) (Figure 2).

### Validation of NOLUS in the Testing Dataset

The testing dataset was composed of 514 HR+/HER2-negative tumor samples from 3 independent studies (HCB, IBIMA and CBM). The proportion of non-luminal disease here was 6.2% (33/514). NOLUS as a continuous variable was found significantly associated with non-luminal disease (*p* < 0.01) with an AUC 0.902 (Figure 2). The proportion of non-luminal tumors in NOLUS-positive and NOLUS-negative groups was 76.9% (56.4–91.0) and 2.6% (1.4–4.5), respectively (*p* < 0.01). The sensitivity was 59.3 and the specificity was 98.7%. To identify only HER2-E, the sensitivity was 42.8 and the specificity was 96.0%. To identify only Basal-like, the sensitivity was 53.9 and the specificity was 99.0%.

### NOLUS in All Datasets

**Figure 3:**
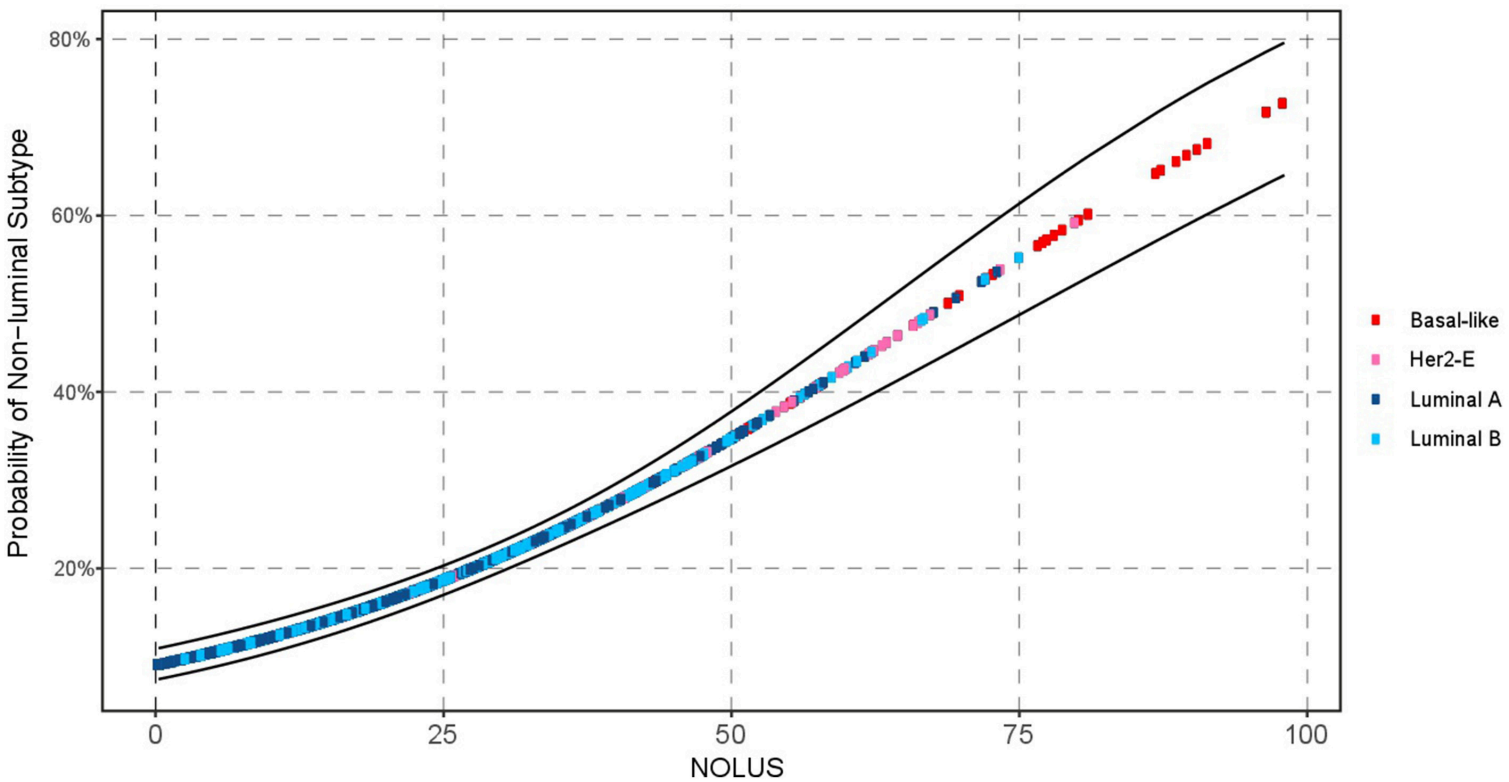
Probability of non-luminal disease as a function of NOLUS in all patients.

We explored NOLUS in all datasets combined. The odds of being non-luminal subtype increase 6.8% for every point increase (OR = 1.068, 95% CI 1.06–1.08, *p* < *0.001*). The rates of non-luminal in NOLUS-negative and NOLUS-positive were 6.52 and 60.24%, respectively (Adjusted OR = 23.82, 95% CI 13.97–40.61, *p* < 0.001) (Figure 3).

Finally, the model was validated using 10-fold cross validation. The data was separated into 10 sets, each set containing 10% of the data. For each validation round, 9 sets were used as training data, and the other set was used as testing data to validate the model using the linear discriminant analysis method. The accuracy of the model with 10-fold cross-validation was 0.97 (Cohen's kappa coefficient = 0.83).

## Discussion

In this study, we aimed to identify a pathology-based model that is easy, fast and with the potential to be widely implemented to identify non-luminal disease within HR+/HER2-negative breast cancer when gene expression data is not available. The main reasons are that there is accumulating evidence that non-luminal disease within HR+/HER2-negative disease represents a distinct biological and clinical entity (14) that deserves substantial attention and that gene expression-based assays are not always readily available in daily clinical practice. To our knowledge, this is the first report to attempt to derive a pathology-based predictive model to identify PAM50 non-luminal disease within HR+/HER2-negative disease.

The importance of intrinsic subtyping was highlighted in one of the most complete molecular characterization studies that has ever been performed in breast cancer (4). In this study, led by The Cancer Genome Atlas Project (TCGA), more than 500 primary breast cancer were extensively profiled at the DNA (i.e., methylation, chromosomal copy-number changes and somatic and germline mutations), RNA (i.e., miRNA and mRNA expression) and protein (i.e., protein and phosphor-protein expression) levels using the most recent technologies (4). In a particular analysis of over 300 primary tumors, 5 different data-types (i.e., all except DNA mutations) were combined together in a cluster of clusters in order to identify how many biological homogenous groups of tumors one can identify in breast cancer. The consensus clustering results showed the presence of 4 main entities of breast cancer but, more importantly, these 4 entities were found to be very-well recapitulated by the 4 main intrinsic subtypes (Luminal A, Luminal B, HER2-E, and Basal-like) as defined by mRNA expression only (3, 5, 6, 36, 38–40). Overall, these results suggest that intrinsic subtyping captures the vast majority of the biological diversity occurring in breast cancer.

Although the incidence of the Basal-like and HER2-E subtypes within HR+/HER2-negative tumors is below 10% in the primary disease setting (4), current evidence suggest that this frequency is much larger in the advanced/metastatic setting, specially following endocrine treatment (14). The increase proportion of the HER2-E subtype in the metastatic setting may be due to setting selection, a change in the biology of the tumor due to the inherent evolution of the tumor or the effects of the treatment, or a combination of both. Current evidence supports this latter possibility. Patients with early HR+/HER2-negative/HER2-E breast cancer have a higher probability of relapse than luminal disease. Therefore, it is likely that a given population of patients with metastatic disease is more enriched for the HER2-E subtype compared to patients with early breast cancer. Moreover, using 123 pairs of primary vs. metastatic tumor samples with a high proportion of HR+/HER2-negative tumors, Cejalvo et al. (28) showed that the HER2-E signature and HER2-E subtype are enriched in the metastatic samples compared to primary tumors. For example, 13% of primary Luminal A and B tumors were identified as HER2-E in the relapsed tumor sample. Overall, the proportion of HER2-E tumors in primary vs. metastatic was 11.4 vs. 22%, respectively. Moreover, in a retrospective analysis of tumor samples from the BOLERO-2 study, where patients with HR+/HER2-negative advanced disease resistant to an aromatase inhibitor, the proportion of HER2-E in primary vs. metastatic tumors was 19 vs. 32% (41). Recently, gene expression data from the PALOMA-2 clinical trial have been presented (21, 22). In this retrospective analysis, which included 68% (445/666) of the tumors of both primary and metastatic tumors within the clinical trial population, the HER2-E population represented 19 and the Basal-like population represented 1%.

The prognostic value of the Basal-like and HER2-E intrinsic subtypes in HR+/HER2-negative breast cancer has been evaluated in several studies (22–24). For example, intrinsic subtyping performed in a cohort of 1,380 patients with ER+ early breast cancer treated with 5 years of adjuvant tamoxifen-only (23) demonstrated the presence of a 7% of non-Luminal disease. These patients showed a statistically significant worse outcome compared to Luminal A subpopulation. The prognostic value of the HER2-E intrinsic subtype has been evaluated also in 3 retrospective studies involving HR+/HER2-negative metastatic patients (22, 24, 41). In the EGF30008 Phase III clinical trial, intrinsic subtyping was performed in a cohort of 821 patients with HR-positive disease (644 HER2-negative and 157 HER2+) treated in the first-line metastatic setting with either letrozole or letrozole plus lapatinib (24). Patients with HER2-E and Basal-like disease showed worse outcome in terms of progression free survival (PFS) and overall survival (OS) compared to Luminal A disease regardless of the HER2 status and treatment. Compared with the Luminal A subtype, the non-luminal subtypes showed a significantly decreased PFS independently of other clinical-pathological variables. Patients with HER2-E, and Basal-like subtypes had a 2.87, and 2.26 times higher risk of tumor progression, respectively. Median PFS differed across the intrinsic subtypes: Luminal A (16.9 months), Luminal B (11.0 months), HER2-E (4.7 months), and Basal-like (4.1 months). In the second study, PAM50 was performed in 261 tumor samples from the BOLERO-2 phase III trial (41). The subtype distribution was: 46.7% Luminal A, 21.5% HER2-negativeE, 15.7% Luminal B, 14.2% Normal-like and 1.9% Basal-like. Non-luminal disease was independently associated with poor PFS and OS compared to the luminal subtypes. In the third study, PAM50 was performed in 465 tumor samples from the PALOMA-2 phase III trial. Both non-luminal subtypes were associated with worse PFS compared to Luminal A subtype. These results support that non-luminal HR+/HER2-negative tumors are aggressive and require novel therapeutic approaches.

The ability of the Basal-like and HER2-E subtype to predict benefit from anti-estrogen therapy has been evaluated in the neoadjuvant setting. In the Z1031 neoadjuvant trial (16) within ER+/HER2-negative disease, patients with HER2-E or Basal-like disease had persistently high surgical Ki67 levels (20%) after 4–6 months of treatment with an aromatase inhibitor, consistent with high-level estrogen-independent growth. In another retrospective study of 112 postmenopausal women with stages I–IIIB ER+ early breast cancer before and after 2-weeks' anastrozole treatment in a neoadjuvant trial, patients with HER2-E subtype (*n* = 9 [8.0%]) or Basal-like subtype (*n* = 3 [2.7%]) showed a poorer Ki67 response (mean Ki-67 change of−50.7 and +15.3%) compared to Luminal A or B subtypes (mean Ki-67 change of−75%). Interestingly, this study also profiled post-treatment samples. As expected, the vast majority of Luminal A samples (31/32, 97%) continued being Luminal A. However, although the majority of Luminal B tumors became Luminal A (9/17, 53%), 12% (2/17) became HER2-E. Overall, this data, together with the poor PFS of the HER2-E subtype following endocrine therapy in EGF30008, BOLERO-2 and PALOMA 2 trials (22, 24, 41), suggest that both non-luminal subtypes within HR-positive disease might not benefit substantially from anti-estrogen therapy.

The ability of the Basal-like and HER2-E subtype to predict benefit from palbociclib has been recently evaluated in 465 samples of the PALOMA-2 study (22). The increase in median PFS in the HER2-E subtype was modest (2.8 months), compared to the increase in median PFS of 13.4 and 8.6 months in Luminal A and B subtypes, respectively. Regarding Basal-like, only 1 patient was identified and progressed at 6.4 months following letrozole plus palbociclib. This data suggest that non-luminal subtypes do not benefit much from CDK4/6 inhibition. In the neoadjuvant setting, Ma and colleagues conducted the NEOPALANA clinical trial with anastrozole and palbociclib. Two non-luminal tumors were identified by PAM50 (1 HER2-E and 1 Basal-like) and, interestingly, none of the 2 patients responded to the combined treatment (17).

The ability of the Basal-like and HER2-E subtype to predict chemotherapy sensitivity within HR+/HER2- disease has been evaluated in the neoadjuvant setting. In one study, we evaluated the pathological complete response (pCR) rated in 451 patients with HR+/HER2-negative disease treated with standard multi-agent neoadjuvant chemotherapy (42). The pCR rates in the non-luminal subtype was 23.2% compared to 15% in Luminal B and 5% in Luminal A tumors. In another neoadjuvant study, Prat and colleagues evaluated the residual cancer burden (RCB) 0/1 rates of the intrinsic subtypes in 180 patients with HR+/HER2-negative disease treated with anthracycline/taxane-based chemotherapy (18). Concordant with the first study, the RCB0/1 rates were higher in the non-luminal subtypes (38.1%) compared to Luminal B (20.0%) and Luminal A (9.3%). Overall, this data suggests that within HR+/HER2-negative disease, non-luminal tumors are highly chemo-sensitive.

Our study has several limitations worth noting. For example, determination of ER, PR and Ki67 was not performed centrally in a single lab and, in 2 studies, IHC data was obtained from local pathology reports. In addition, each study used different pathology-based assays. Although this heterogeneity is a limitation, its effects must not be large since the proportion of non-luminal disease across studies was similar and the fact that NOLUS was able to predict non-luminal disease in both the training and testing sets with similar performance. Another limitation is that NOLUS is not a standardized assay; thus, analytical validity is lacking. However, the biomarkers that compose NOLUS (i.e., ER, PR, and Ki67) have not been standardized; thus, NOLUS will suffer from lack of standardization as well. Another aspect is that we did not aim to derive a model that could further distinguish Basal-like from HER2-E subtypes within non-luminal disease. The main reason is that at this point it is unclear what are the clinical implications of each of these entities both from a prognostic and predictive point of view. However, as more data is gathered, NOLUS could be updated in the future to further distinguish these 2 non-luminal subtypes. Finally, we do not provide clinical validation of the NOLUS predictor.

To conclude, NOLUS is a tool that, in the absence of gene expression-based assays, may help identify non-luminal disease within HR+/HER2-negative breast cancer. Overall, the data clearly suggest that both non-luminal subtypes provide additional prognostic and predictive information beyond HR and HER2 status and may support more informed treatment decisions (1). For example, to identify patients who are not good candidates for endocrine therapy alone. Pivotal and large studies evaluating prognosis and treatment benefits can now apply NOLUS and further define the clinical validity and clinical utility of this biomarker.

## Author Contributions

All authors participated in the design and/or interpretation of the reported results and participated in the acquisition and/or analysis of data. In addition, all authors participated in drafting and/or revising the manuscript and provided administrative, technical, or supervisory support.

### Conflict of Interest Statement

AP reports consulting and lecture fees from Nanostring Technologies outside the submitted work. **Á**R-L reports Clinical Research from Amgen, Astra Zeneca, Boehringer-Ingelheim, GSK, Novartis, Pfizer, Roche/Genentech, Eisai, Celgene, and Pierre Fabre and Advisory Boards and Consulting from Novartis, Pfizer, Roche/Genentech, Eisai, and Celgene, outside the submitted work. GP reports lecture fees from Nanostring Technologies and Clinical Research funds from Astrazeneca, outside the submitted work. The remaining authors declare that the research was conducted in the absence of any commercial or financial relationships that could be construed as a potential conflict of interest.
