# Supplementary material for: A Pathology-Based Combined Model to Identify PAM50 Non-luminal Intrinsic Disease in Hormone Receptor-Positive HER2-Negative Breast Cancer
Source: Front Oncol. 2019 Apr 26;9:303. doi: 10.3389/fonc.2019.00303 (PMC6498671; doi:10.3389/fonc.2019.00303)
Supplement: Supplementary file 1 [file Table_1.DOCX]

**Table S1.** Logistic regression analyses of non-luminal disease.

|  |  |  |  | **Univariate Analysis** | | | |  | **Multivariable Analysis** | | | |
| --- | --- | --- | --- | --- | --- | --- | --- | --- | --- | --- | --- | --- |
| **Variables** | ***N*** | **Non Luminal** |  | **OR** | **Lower 95%** | **Upper 95%** | ***P*** |  | **OR** | **Lower 95%** | **Upper 95%** | ***P*** |
| **Study** |  |  |  |  |  |  |  |  |  |  |  |  |
| PETx | 56 | 5.3 % |  | 1 | - | - | *-* |  | 1 | - | - | *-* |
| GEICAM/2009-03 | 50 | 14% |  | 2.328 | 0.58 | 9.21 | *0.229* |  | 4.372 | 1.00 | 19.02 | *0.049* |
| GEICAM/2012_09 | 173 | 2.9% |  | 0.542 | 0.12 | 2.34 | *0.413* |  | 0.666 | 0.15 | 3.02 | *0.598* |
| GEICAM/9906 | 531 | 14.7% |  | 3.036 | 0.93 | 9.95 | *0.067* |  | 4.623 | 1.33 | 16.01 | *0.016* |
| Neoeribulin | 93 | 12.9% |  | 2.588 | 0.70 | 9.59 | *0.155* |  | 2.778 | 0.66 | 11.65 | *0.163* |
| **IHQ** |  |  |  |  |  |  |  |  |  |  |  |  |
| Estrogen receptor (%) | 903 | 11.5% |  | 0.971 | 0.96 | 0.97 | *<0.001* |  | 0.976 | 0.97 | 0.98 | *<0.001* |
| Progesterone receptor (%) | 903 | 11.5% |  | 0.984 | 0.98 | 0.98 | *<0.001* |  | 0.985 | 0.98 | 0.99 | *<0.001* |
| Ki67 (%) | 903 | 11.5% |  | 1.017 | 1.01 | 1.03 | *0.002* |  | 1.015 | 1.00 | 1.03 | *0.042* |
